# Reduction in HbA1c using professional flash glucose monitoring in insulin-treated type 2 diabetes patients managed in primary and secondary care settings: A pilot, multicentre, randomised controlled trial

**DOI:** 10.1177/1479164119827456

**Published:** 2019-07-04

**Authors:** Ramzi A Ajjan, Neil Jackson, Scott A Thomson

**Affiliations:** 1 St James’s University Hospital, Leeds Teaching Hospitals NHS Trust and LIGHT Laboratories: Leeds Institute of Cardiovascular and Metabolic Research, University of Leeds, Leeds, UK; 2 Pound Hill Medical Group, Crawley, UK; 3 Atherstone Surgery, Atherstone, UK

**Keywords:** Continuous glucose monitoring, flash glucose monitoring, HbA1c, hypoglycaemia, hyperglycaemia, glycaemic variability

## Abstract

**Aim:**

Analyse the effects of professional flash glucose monitoring system (FreeStyle Libre Pro™) on glycaemic control in insulin-treated type 2 diabetes.

**Methods:**

Primary (n = 17) and secondary care centres (n = 5) randomised 148 type 2 diabetes patients into three groups: (A) self-monitoring of blood glucose (n = 52), (B) self-monitoring of blood glucose and two Libre Pro sensor wears (n = 46) or (C) self-monitoring of blood glucose and four sensor wears (n = 50). Primary endpoint was time in range (glucose 3.9–10 mmol/L) within group C comparing baseline with days 172–187. Predefined secondary endpoints included HbA1c, hypoglycaemia and quality of life measures analysed within and between groups (clinicaltrials.gov, NCT02434315).

**Results:**

In group C, time in range in the first 14 days (baseline) and days 172–187 was similar at 15.0 ± 5.0 and 14.1 ± 4.7 h/day (mean ± SD), respectively, (p = 0.1589). In contrast, HbA1c reduced from baseline to study end within group C by 4.9 ± 8.8 mmol/mol (0.44% ± 0.81%; *p* = 0.0003). HbA1c was also lower in group C compared with A at study end by 5.4 ± 1.79 mmol/mol (0.48% ± 0.16%; p = 0.0041, adjusted mean ± SE), without increased time in hypoglycaemia (*p* = 0.1795). Treatment satisfaction scores improved in group C compared with A (*p* = 0.0225) and no device-related serious adverse events were reported.

**Conclusions:**

Libre Pro can improve HbA1c and treatment satisfaction without increasing hypoglycaemic exposure in insulin-treated type 2 diabetes individuals managed in primary/secondary care centres.

## Introduction

Improving glycaemic control remains a key target to reduce complications in individuals with diabetes.^1–3^ However, lowering glucose levels can be challenging, with less than 65% of individuals with type 2 diabetes reaching optimal HbA1c targets,^4,5^ decreasing further to 24% in those taking insulin.^4^ An important barrier for lowering glucose levels is the precipitation of hypoglycaemia, a potentially life-threatening complication of insulin therapy.^1^

Continuous glucose monitoring (CGM) is an established method for improving glucose levels and reducing the risk of hypoglycaemia in type 1 diabetes.^6^ Although the evidence for the benefits of CGM in type 2 diabetes is generally limited,^7–12^ current guidance endorses the use of retrospective glucose data and intermittent sensor application in the management of insulin-treated type 2 diabetes^13,14^ However, the ability of primary care clinicians to embrace complex glucose monitoring strategies has been questioned^15,16^ despite the majority of clinical management for type 2 diabetes occurring in primary rather than secondary care. For professional CGM technology to be viable in primary care, implementation must be straightforward with glucose data reports that can be rapidly generated and interpreted to aide communication with patients and therapy adjustments. Therefore, the aim of this study was to understand the role of new professional sensor technology and retrospective glucose data in improving glycaemic outcomes using real-life settings with the majority of participants recruited from primary care centres.

## Methods

We used a blinded professional sensor-based glucose monitoring system to perform retrospective glucose data analysis (FreeStyle Libre Pro™; Abbott Diabetes Care, Witney, Oxon, UK). The sensor, worn on the upper arm, is factory calibrated and requires no participant or healthcare professional intervention during the 14-day wear. The pro-reader is retained by the healthcare professional and data are not visible to participants or investigators during sensor wear. The sensor automatically captures and stores glucose data every 15 min (96 glucose readings/day). At follow-up visits, scanning the sensor will wirelessly transfer glucose data to the pro-reader. Summary glucose reports (including ambulatory glucose profile) for review and analysis by the healthcare professional are generated using the system software.^17^

### Study design and participants

This pilot, prospective, randomised controlled trial was conducted at 18 general primary healthcare centres and five secondary care diabetes centres in England (total centres n = 23). Eligible participants were ⩾18 years of age with type 2 diabetes treated with insulin therapy for at least 6 months and an HbA1c level between 58 and 108 mmol/mol (7.5%–12.0%). Exclusion criteria included use of animal insulin, total daily dose of insulin (TDD) > 1.75 iu/kg on study entry, insulin pump therapy, CGM use in the last 6 months (including professional CGM), steroid therapy for any condition, pregnant or planning pregnancy, allergy to medical grade adhesives or considered unsuitable to participate by the investigator.

Ethics Committee approval was granted prior to the study and all procedures were in accordance with the Helsinki Declaration of 1964, as revised in 2013. Informed, written consent was obtained from all participants in the study. This trial is registered with ClinicalTrials.gov (NCT02434315).

### Randomisation and masking

At each centre, participants were randomly assigned to one of three groups for clinical review with readings from self-monitoring of blood glucose (SMBG) as per standard care (group A, control arm), additional use of two Libre Pro sensor wears (group B) or four Libre Pro sensor wears (group C). Randomisation was by permuted block with varying block sizes in a 1:1:1 ratio using randomly generated opaque, sealed envelopes. Participants and investigators were not masked to group allocation.

### Procedures

**Figure 1.**
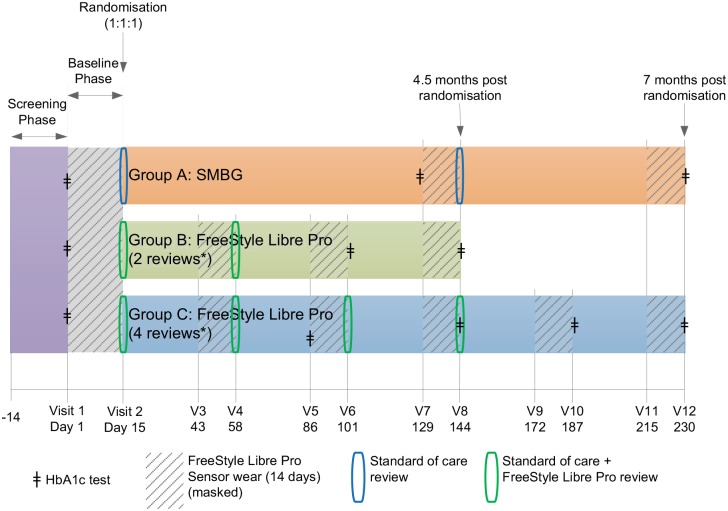
Trial design. Following consent, screening and enrolment, all participants had a professional flash sensor applied to the upper arm for the 14-day baseline period. Participants with baseline sensor data of ⩾500 sensor glucose readings were randomised to one of the three study groups as shown.

Following consent, screening and enrolment, all participants had a professional flash sensor applied to the upper arm for the 14-day baseline period. Glucose management was supported by their usual regimen of SMBG using their personal device. During each sensor wear, participants were asked to record SMBG results, meals, insulin doses and any physical activity in a diary. Participants with baseline sensor data of ⩾500 sensor glucose readings were randomised to one of the three study groups detailed above (Figure 1). Post randomisation, sensor glucose data were uploaded for intervention participants (group B and group C) and glucose reports (e.g. the ambulatory glucose report) were generated using the system software.^17^ This information was used by the healthcare professional to support an individualised glucose management plan while participants continued SMBG testing during the treatment phase. Intervention groups B and C had one and three further sensor wear periods, respectively, 1 month apart, followed by review with the healthcare professional (Figure 1).

For group A (control) participants, only SMBG results were used by the healthcare professional to support an individualised glucose management plan, as per standard diabetes care in the United Kingdom. This included continuing to use their personal device for SMBG testing during the treatment phase (Figure 1).

There were no pre-set insulin algorithm or treatment protocols used in the study. HbA1c was measured in all participants using local laboratory testing (Figure 1).

Patient-recorded outcome measures for the Diabetes Treatment Satisfaction Questionnaire (DTSQ)^18^ were assessed at baseline and at the penultimate visit for each study arm (Figure 1). A user questionnaire (intervention participants) and a healthcare professional user questionnaire (clinicians) were completed when intervention group participants completed their second clinical review for the study (day 58).

### Outcomes

The primary outcome was change in sensor derived time in range (glucose 3.9–10.0 mmol/L [70–180 mg/dL]) within intervention group C between the baseline phase and penultimate sensor wear (days 172–187). Pre-specified secondary endpoints included difference in HbA1c and sensor derived glycaemic measurements, TDD, insulin regimen changes, body mass index (BMI)/weight and self-reported SMBG frequency, analysed within and between groups. Glycaemic measurements included number and duration of hypoglycaemic events (<3.9 mmol/L, [70 mg/dL]), number and duration of hyperglycaemic events (>10 mmol/L, [180 mg/dL]; >13.3 mmol/L [240 mg/dL]), mean glucose and glucose variability measurements. An event was defined as at least two consecutive readings at 15-min intervals, outside the predefined sensor glucose range (an event ended with one reading returning within the predefined range).

Pre-specified sub-group analysis of endpoints included the location of diabetes management, type of insulin regimen and glycaemic measures for day- (06.00–23.00) and night-time (23.00–06.00). Safety endpoints incorporated monitoring of all adverse events throughout the study including severe hypoglycaemia^19^ and sensor insertion or sensor wear–related symptoms. Validated quality of life questionnaires investigated patient satisfaction with the glucose monitoring strategy and health care questionnaire addressed satisfaction of the professional team looking after the patient.

### Statistical analysis

Differences within group, including the primary endpoint analysis, were assessed by a paired t-test. Missing values were imputed by last observation carried forward. This included the baseline value if no measurements after baseline were available. Differences between groups were considered by analysis of covariance on the baseline values with site as a covariate. Confidence intervals were calculated for the group least squares mean of each measure and the difference between group least squares means. To detect a change of 1.5 h/day in time in range (TIR; 3.9–10.0 mmol/L) within group C with a power of 80% (at p < 0.05), a total of 40 individuals are needed, based on SD of TIR of 3.5 h/day.^20^ Assuming a drop out of 20%, we aimed to recruit 48 participants into each study group.

Data analysis was performed by qualified statisticians at Abbott Diabetes Care. Version 9·2 of SAS (or higher) was used for all analyses.

## Results

We enrolled 175 participants between 27 April 2015 and 13 October 2015; 148 participants were eligible for randomisation following the baseline phase and were assigned to control group A (n = 52), intervention group B (n = 46) or intervention group C (n = 50); 70.9% (n = 105) of participants were from primary care. Baseline characteristics are shown in Table 1.

**Table 1.** Baseline characteristics of participants.

	Control group A (n = 52)	Intervention group B (n = 46)	Intervention group C (n = 50)	All (N = 148)
Age (years)	65.0 ± 11.2	63.9 ± 10.7	61.7 ± 11.1	63.6 ± 11.0
Weight (kg)	92.1 ± 16.7	94.5 ± 19.6 (n = 44)	95.5 ± 18.6	94.0 ± 18.2 (n = 146)
BMI (kg/m^2^)	32.1 ± 5.5	32.3 ± 5.8 (n = 44)	33.2 ± 6.5	32.6 ± 5.9 (n = 146)
Insulin therapy				
Basal only	21 (40.4%)	24 (52.2%)	21 (42.0%)	66 (44.6%)
Basal/bolus	26 (50.0%)	19 (41.3%)	25 (50.0%)	70 (47.3%)
Biphasic	5 (9.6%)	3 (6.5%)	4 (8.0%)	12 (8.1%)
Duration of insulin use (years)	9.5 ± 6.5	7.3 ± 4.8	8.2 ± 6.3	8.4 ± 6.0
Duration of current insulin regimen (years)	8.0 ± 5.7	5.8 ± 4.1	6.5 ± 5.0	6.8 ± 5.0
Self-reported SMBG frequency per day	2.3 ± 1.3	2.2 ± 1.6	2.2 ± 1.5	2.3 ± 1.5
Screening HbA1c (%)	8.7 ± 1.0	8.7 ± 1.1	8.7 ± 0.8 (n = 49)	8.7 ± 1.0 (n = 147)
Screening HbA1c (mmol/mol)	71.4 ± 11.3	71.5 ± 12.2	71.3 ± 9.3 (n = 49)	71.4 ± 10.9 (n = 147)
Male	28 (53.8%)	28 (60.9%)	28 (56.0%)	84 (56.8%)
Female	24 (46.2%)	18 (39.1%)	22 (44.0%)	64 (43.2%)
Employment status				
Full-time	16 (30.8%)	9 (19.6%)	12 (24.0%)	37 (25.0%)
Part-time	4 (7.7%)	3 (6.5%)	4 (8.0%)	11 (7.4%)
Full-time student	0	0	2 (4.0%)	2 (1.4%)
Not employed/retired	32 (61.5%)	32 (69.6%)	32 (64.0%)	96 (64.9%)
Other	0	2 (4.3%)	0	2 (1.4%)
Race				
White	50 (96.2%)	44 (95.7%)	44 (88.0%)	138 (93.2%)
Asian	1 (1.9%)	2 (4.3%)	4 (8.0%)	7 (4.7%)
Mixed	1 (1.9%)	0	1 (2.0%)	2 (1.4%)
Black	0	0	1 (2.0%)	1 (0.7%)

Data are n (%) or mean ± SD. There were no statistically significant differences between groups. BMI: body mass index; SMBG: self-monitoring of blood glucose; SD: standard deviation.

**Figure 2.**
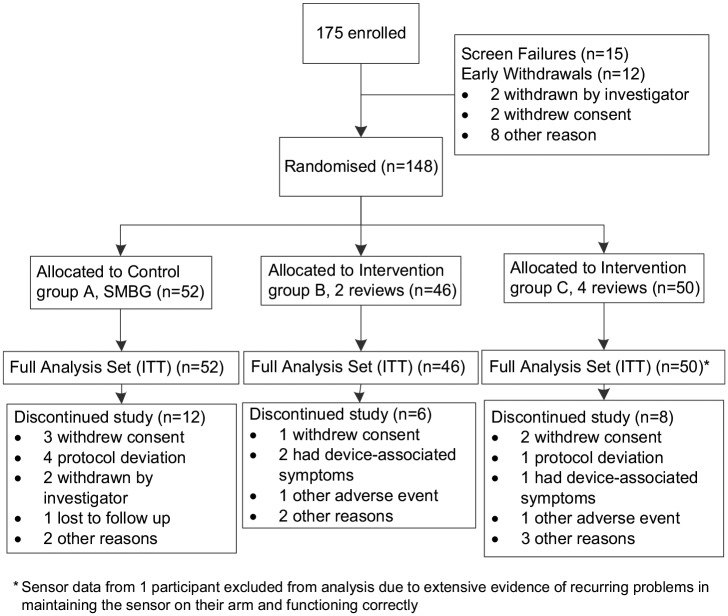
Trial profile demonstrating withdrawal rate.

The full analysis set included all 148 randomised participants. There were 27 withdrawals before randomisation and 26 participants discontinued the study after randomisation (Figure 2).

### Time in range

**Figure 3.**
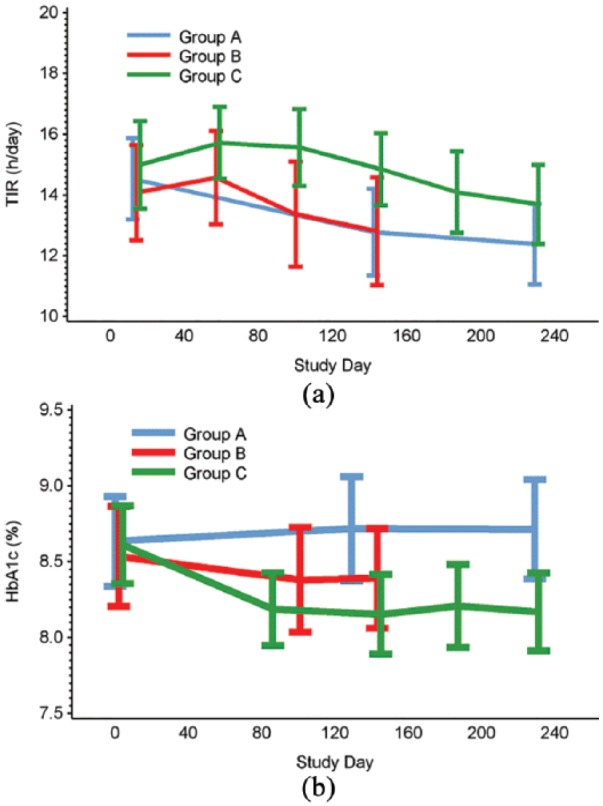
Time in (a) glucose range 3.9–10 mmol/L and (b) HbA1c, during the baseline and treatment phases.

There was no change in TIR (sensor glucose 3.9–10.0 mmol/L [70–180 mg/dL]) within group C comparing baseline (15.0 ± 5.0 h/day [mean ± SD]) with days 172–187 (14.1 ± 4.7, p = 0.1589). No difference was found compared with the control group at study end (days 215–230; Table 2, Figure 3).

**Table 2.** Glycaemic and glucose variability measures.

Control group A (n = 52) Intervention group C (n = 49) Glycaemic measure		Baseline mean (SD)		Day 215–230 mean (SD)		Difference in adjusted means vs control (SE)	*p* vs control
		Intervention group C	Control group	Intervention group C	Control group		
Glucose 3·9–10.0 mmol/L (70–180 mg/dL)	Time, hours	15.0 (5.0)	14.5 (4.8)	13.7 (4.5)	12.4 (4.8)	0.96 (0.77)	0.2193
HbA1c (mmol/mol)		70.7 (9.8)	70.8 (11.6)	65.8 (9.8)	71.8 (12.8)	−5.4 (1.79)	0.0041
HbA1c (%)		8.6 (0.9)	8.6 (1.1)	8.2 (0.9)	8.7 (1.2)	−0.48 (0.16)	0.0041
Glucose <3·9 mmol/L (70 mg/dL)	Time, hours	1.66 (1.64)	1.77 (2.08)	1.76 (2.35)	1.33 (1.51)	0.51 (0.38)	0.1795
	Events	0.74 (0.61)	0.75 (0.70)	0.70 (0.77)	0.61(0.61)	0.09 (0.13)	0.4660
Glucose >13·3 mmol/L (240 mg/dL)	Time, hours	2.74 (3.64)	2.71 (3.35)	3.39 (3.69)	4.60 (4.85)	−1.03 (0.70)	0.1458
	Events	0.87 (0.77)	0.92 (0.81)	1.12 (0.79)	1.25 (0.75)	−0.12 (0.13)	0.3640
Glucose > 10.0 mmol/L (180 mg/dL)	Time, hours	7.35 (5.42)	7.70 (5.18)	8.55 (5.32)	10.29 (5.41)	−1.50 (0.89)	0.0970
	Events	1.89 (0.75)	1.82 (0.71)	2.07 (0.82)	2.11 (0.70)	−0.10 (0.14)	0.4891
Mean glucose mmol/L		8.7 (2.1)	8.7 (1.9)	9.1 (2.2)	9.9 (2.6)	−0.71 (0.41)	0.0866
SD glucose mmol/L		3.0 (0.81)	3.1 (0.91)	3.1 (0.87)	3.4 (0.84)	−0.13 (0.12)	0.2510
CV glucose (%)		34.4 (7.7)	35.3 (7.7)	34.8 (6.2)	35.0 (8.0)	0.29 (0.92)	0.7547
LGI		1.45 (1.25)	1.53 (1.57)	1.39 (1.56)	1.15 (1.22)	0.29 (0.27)	0.2851
HGI		7.20 (6.04)	7.41 (5.52)	8.42 (6.16)	10.78 (8.36)	−1.98 (1.25)	0.1180
SD of glucose rate of change (mg/dL/min)		0.73 (0.14)	0.72 (0.14)	0.75 (0.15)	0.78 (0.14)	−0.04 (0.02)	0.0545
CONGA	1 h (mmol/L)	1.84 (0.40)	1.80 (0.37)	1.92 (0.44)	1.99 (0.38)	−0.10 (0.06)	0.1076
	2 h (mmol/L)	2.80 (0.64)	2.77 (0.57)	2.95 (0.72)	3.06 (0.62)	−0.12 (0.10)	0.2300
	4 h (mmol/L)	3.72 (0.95)	3.73 (0.86)	3.94 (1.07)	4.13 (0.90)	−0.16 (0.14)	0.2754

SD: standard deviation, CV: coefficient of variation, LGI: low glucose index, HGI: high glucose index, CONGA: continuous overall net glycaemic action. Time and events are per day.

There was no change in TIR in group B compared with the control group at days 129–144 (0.3 ± 0.86 h/day [adjusted mean ± standard error (SE)], *p* = 0.6891).

### HbA1c measurements

HbA1c level reduced in group C (from 70.7 ± 9.8 mmol/L mean ± SD [8.6% ± 0.9%] at baseline to 65.8 ± 9.8 mmol/L [8.2% ± 0.9%] at day 230), while this did not change in the control group (70.8 ± 11.6 mmol/mol [8.6% ± 1.1%] at baseline to 71.8 ± 12.8 [8.7% ± 1.2%]), Table 2, Figure 3). A significant difference was also detected comparing HbA1c in group C with A at study end (adjusted mean ± SE: –5.4 ± 1.8 [–0.48% ± 0.16%], *p* = 0.0041).

There was no difference in HbA1c in group B compared with the control group (–2.9 ± 1.9 mmol/mol [–0.26% ± 0.17%], *p* = 0.1331, day 144).

### Other glycaemic parameters

**Figure 4.**
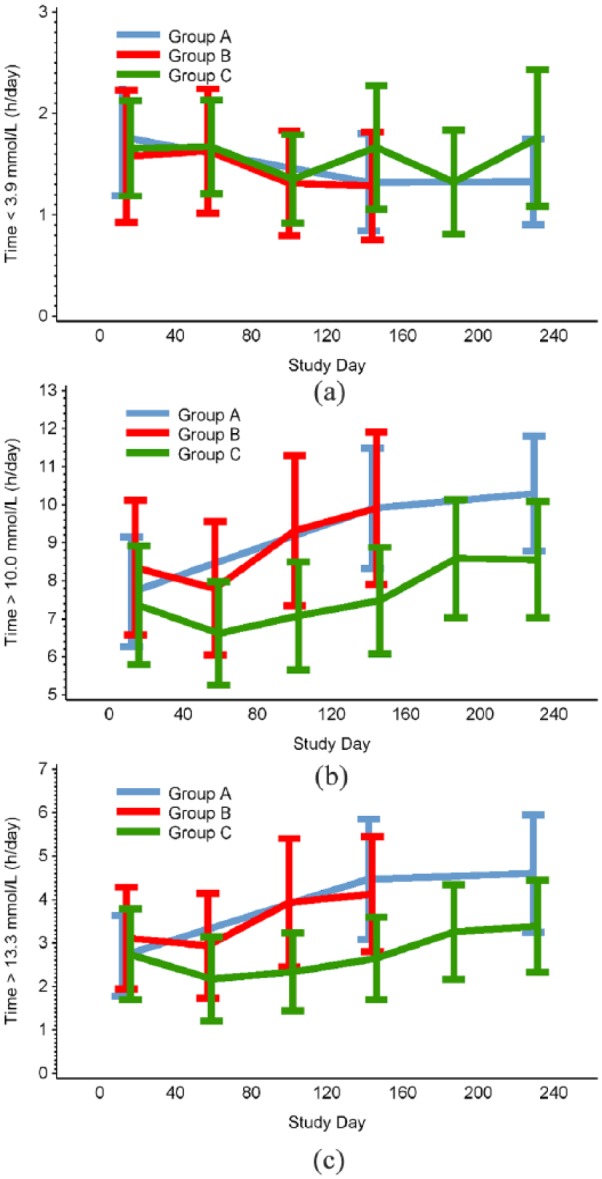
Time in (a) hypoglycaemia <3.9 mmol/L, (b) hyperglycaemia >10 mmol/L and (c) hyperglycemia >13.3 mmol/L, during the baseline and treatment phases.

At study end (day 215–230), time in hyperglycaemia >180 mg/dL (> 10.0 mmol/mol) was 8.6 ± 5.3 h/day (mean ± SD) in group C and 10.3 ± 5.4 h/day in group A (control group) but this difference of –1.50 ± 0.89 h/day (adjusted mean ± SE) failed to reach statistical significance (*p* = 0.097, Table 2, Figure 4).

Hypoglycaemic measures (time, number of episodes and AUC) <3.9 mmol/L (70 mg/dL) were unaffected in group C compared with group A (*p* = 0.1795, *p* = 0.4660 and *p* = 0.1098, respectively; Table 2, Figure 4).

A trend towards lower mean glucose in group C compared with group A was shown (*p* = 0.0866). The difference in SD of glucose rate of change for group C compared to group A was nearing significance (*p* = 0.0545), whereas other measures of glycaemic variability were unchanged (Table 2).

There was no observed change in time in hyperglycaemia (>180 mg/dL [>10.0 mmol/mol], >13.3 mmol/L [240 mg/dL]; *p* = 0.6404, *p* = 0.3038, respectively), hypoglycaemia (<3.9 mmol/L [70 mg/dL], *p* = 0.8530) and mean glucose (*p* = 0.3350) for group B compared with the control group at days 129–144.

In group C, 48% of participants who improved their time in hypoglycaemia by ⩾30% also improved their HbA1c by ⩾0.5%, compared with 17% in the control group (*p* = 0.0327).

Pre-specified sub-group analysis showed no difference in TIR within group C or when compared to control participants (group A), for participants managed within primary care (105/148) or in secondary care (43/148).

### Role of insulin regimen

Pre-specified sub-group analysis of HbA1c level by insulin regimen demonstrated significant improvement at day 230 for group C participants using multiple daily injection (MDI) or biphasic insulin therapy (n = 29) of –0.67% ± 0.19% (adjusted mean ± SE, *p* = 0.0010) compared with group A (n = 31). For participants using basal insulin only, change in HbA1c (0.41% ± 0.39%) was not significant (*p* = 0.3011).

There was no change in the TDD for intervention participants compared with the control group. Five participants changed from a basal only insulin regimen to include bolus or biphasic insulin, two participants from the control group, two from intervention group B and one from intervention group C.

Group C participants showed no difference in change in weight at day 230 compared with controls (0.22 ± 0.95 kg [adjusted mean ± SE]; *p* = 0.8215).

### Treatment satisfaction

**Figure 5.**
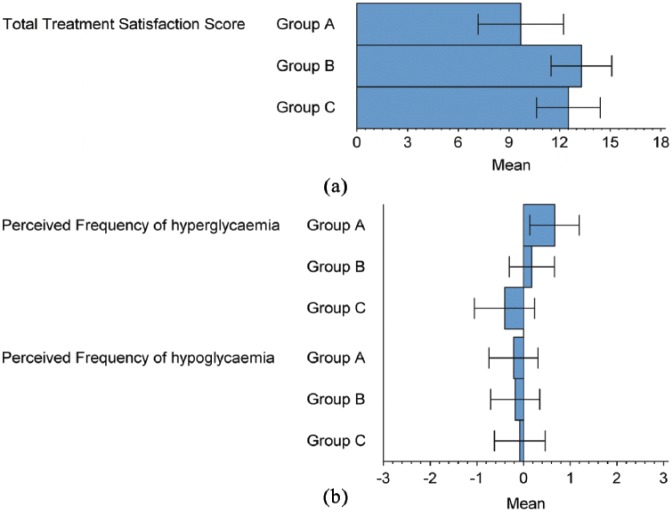
Scores from the DTSQ Questionnaire for (a) total treatment satisfaction score and (b) perceived frequency of hyperglycaemia and hypoglycaemia.

DTSQ scores showed increased overall treatment satisfaction for intervention group B and C participants compared to control (3.45 ± 1.54 [adjusted mean ± SE], *p* = 0.0277 and 3.54 ± 1.52, *p* = 0.0225, respectively, Figure 5).

User (Table 3) and healthcare professional questionnaire (Supplemental Tables S1–S3) responses to using the device and the glucose reports were equally positive. Intervention participants (n = 85) reviewing glucose reports with their healthcare professional agreed the reports were easy to understand (94%) and by using them the healthcare professional was able to explain how current therapies worked (99%), identify abnormalities in glucose levels (96%) and the rationale for treatment changes (98%). As a consequence, understanding of self-management needs improved in all participants (100%), thereby aiding learning about glucose control (95%) and supporting self-management (93%). Both primary and secondary care healthcare professionals (n = 47) agreed the visual nature of the glucose reports supported effective communication with the patient (96%); were easy to read (91%) and understand (85%) to quickly determine abnormal glucose levels (>80%) and what therapy changes were needed (>77%).

**Table 3.** User questionnaire responses.

Indicate your level of agreement with the following statements:	Strongly agree	Agree	Neither agree or disagree	Disagree	Strongly disagree	No response provided
The sensor was comfortable to wear	60 (70.6%)	21 (24.7%)	1 (1.2%)	1 (1.2%)		2 (2.4%)
The sensor was easy to wear due to its small size	58 (68.2%)	20 (23.5%)	5 (5.9%)	1 (1.2%)	1 (1.2%)	
I believe that other people did not notice that I was wearing the sensor	48 (56.5%)	18 (21.2%)	13 (15.3%)	4 (4.7%)	2 (2.4%)	
While wearing the sensor, I did not feel any discomfort under my skin	61 (71.8%)	17 (20.0%)	4 (4.7%)	2 (2.4%)		1 (1.2%)
The sensor did not get in the way of my daily activities	59 (69.4%)	20 (23.5%)	3 (3.5%)	2 (2.4%)	1 (1.2%)	
The reports were easy to understand	42 (49.4%)	38 (44.7%)	3 (3.5%)	1 (1.2%)		1 (1.2%)
My doctor/nurse was able to use the reports effectively to explain how my current diabetes treatment plan is working	63 (74.1%)	21 (24.7%)			1 (1.2%)	
My doctor/nurse was able to use the reports effectively to indicate areas where my glucose management could be improved	59 (69.4%)	23 (27.1%)	2 (2.4%)			1 (1.2%)
My doctor/nurse was able to use the reports effectively to explain why he or she suggested changes to my glucose management	59 (69.4%)	24 (28.2%)	2 (2.4%)			
My doctor/nurse used the reports to personalise my diabetes treatment plan for me	55 (64.7%)	24 (28.2%)	5 (5.9%)	1 (1.2%)		
I think that it would be useful to repeat sensor wear at intervals to review my progress in managing my diabetes	66 (77.6%)	19 (22.4%)				
Seeing my complete glucose profile helps me to understand my glucose levels	66 (77.6%)	19 (22.4%)				
Seeing my complete glucose profile helps me to manage my diabetes better	59 (69.4%)	20 (23.5%)	6 (7.1%)			
I learned something about my diabetes by reviewing the reports with my doctor/nurse	60 (70.6%)	21 (24.7%)	3 (3.5%)			1 (1.2%)
I would recommend this system to others	67 (78.8%)	18 (21.2%)				

### Adverse events

A total of 248 adverse events were experienced by 102 participants (Table 4). This included 26 serious adverse events for 22 participants; none of which were device-related.

**Table 4.** Adverse events.

	Control group A (n = 52)	Intervention group B (n = 46)	Intervention group C (n = 50)	Not randomised (n = 27)	Total (N = 175)
Participants with adverse or serious adverse events	29 (55.8%)	30 (65.2%)	41 (82.0%)	2 (7.4%)	102 (58.3%)
Number of adverse or serious adverse events	61	64	121	2	248
Participants with serious adverse events	8 (15.4%)	7 (15.2%)	7 (14.0%)	0	22 (12.6%)
Number of serious adverse events	9	9	8	0	26
Participants with hypoglycaemia serious adverse events^a^	1 (1.9%)	0	0	0	1 (0.6%)
Number of hypoglycaemia serious adverse events	1	0	0	0	1
Participants with hypoglycaemia adverse events^a^ (excluding serious)	0	1 (2.2%)	3 (6.0%)	0	4 (2.3%)
Number of hypoglycaemia adverse events (excluding serious)	0	1	3	0	4
Participants with device-related adverse events^b^	0	0	2 (4.0%)	0	2 (1.1%)
Number of device-related adverse events^b^	0	0	2	0	2
Participants with study procedure-related adverse events^b^	0	0	1 (2.0%)	0	1 (0.6%)
Number of study procedure-related adverse events^b^	0	0	1	0	1

Table includes the full analysis set. No serious adverse events were related to the study device or procedure.

a No hypoglycaemic events were related to the device.

b Device and/or study procedure adverse events were reported as ‘possibly related’ to sensor wear: one patient with superficial thrombophlebitis (mild) and one with redness (mild).

One control group participant experienced one severe hypoglycaemic event.^18^ In addition, four intervention participants reported four adverse events related to symptomatic hypoglycaemia. No mild or moderate hypoglycaemic events were related to the study device or procedure.

Two mild adverse events from two intervention participants were possibly related to wearing the sensor (superficial thrombophlebitis, resolved without treatment; redness, partially resolved with non-prescription creams). These all resolved.

There were no diabetic ketoacidosis or hyperosmolar hyperglycaemic state events reported during the study.

### Sensor insertion/wear symptoms

Sensor insertion-site signs and symptoms (n = 42) experienced by 29 participants (36 mild and 6 moderate) were primarily associated with sensor wear; erythema (16), itching (10) and rash (4). Those related to sensor insertion were bleeding (5), bruising (4), pain (2), oedema (1) and induration (0). All resolved quickly, primarily without topical treatment (Supplemental Table S4).

Three intervention participants withdrew due to repeated mild and moderate sensor wear–related symptoms of rash (n = 1), itching (n = 1) and erythema (n = 1)

## Discussion

To our knowledge, this is the first randomised controlled trial investigating the role of Libre Pro in modulating glycaemia in insulin-treated type 2 diabetes managed in both primary and secondary care settings.^11,21–23^

Although the primary endpoint of change in TIR was not achieved, the study showed a significant reduction in HbA1c in the intervention arm associated with improved quality of life measures. The reduction in HbA1c was evident within and between group analyses, suggesting this is not a spurious finding. As sensor glycaemic readings, patterns and trends could not be seen by any participant in the present study and given they were aware their glucose data were monitored, the likely explanation for the discrepancy between TIR and HbA1c is behaviour modification of study participants while wearing the sensor during the baseline phase. Thus, the decreasing trend in TIR over the study duration for participants randomised to the control arm was likely due to these participants returning to their usual lifestyle/behaviours after the baseline phase was completed and not having a sensor glucose data review included in subsequent clinic visits. The extent of the fall in TIR (i.e. worsening glycaemic control) for the control group was unexpected and more than previously observed in a similar population further supporting the notion of a ‘study effect’.^9^

The blinded nature of the study, possibly giving participants less incentive to continue, may have been a factor in the participant drop-out rate of 18%. Although this was slightly higher than observed in studies using unblinded sensors in type 2 diabetes,^9^ it was accounted for in our power calculations (which were based on 20% drop-out rate).

It is documented that lifestyle modification can markedly influence TIR regardless of monitoring technology.^10^ As an isolated clinical marker, TIR has the advantage of reflecting intra and inter-day glycaemic changes^24^ but may be misleading when individuals modify their lifestyle over a short period of time.^25^ Therefore, having HbA1c as an additional glycaemic marker gives invaluable data as to whether failing to improve TIR is indicative of a lack of effect for the intervention.

Type 2 diabetes disease progression is marked by escalating therapies and deteriorating glycaemic control^26^ which may not respond to intensification of insulin regimens.^15^ Studies using professional continuous monitoring in insulin-treated type 2 diabetes have generally failed to report improvement in HbA1c.^7,27,28^ The clinically significant reduction in HbA1c documented in this study, without increasing hypoglycaemic exposure, is a key finding. Moreover, this was achieved in patients treated in both secondary and primary care settings, supporting generalizability of the results. In particular, the large proportion of participants from primary care sites, who had improvement in HbA1c, supports the use of modern glucose monitoring strategies outside secondary care settings.

There was no change in TDD, however, smaller insulin dose changes may have occurred without any noticeable overall change in total insulin.^9^ As sensor glucose data were not visible in real-time, the fall in time in hyperglycaemia is likely a direct result of reviewing and discussing retrospective sensor data with the healthcare professional.^8^ The intrinsic value and advantage of clinical dialogue between patient and clinician supported by ‘pictorial’ examples of glycaemic issues and improvements shown in the glucose reports is the least measurable factor. The benefit of this was evident by improved treatment satisfaction supporting the intermittent use of real-time CGM. In contrast, Beck et al.^10^ found no improvement in treatment satisfaction in a similar population using real-time-CGM over 6 months. The difference between the two studies may be related to easier application of flash technology compared with CGM or may simply be due to differences in study design and population investigated.

To date, the number of days of professional or intermittent glucose sensor wear for studies in insulin-treated type 2 has been varied and is usually less than seven days.^7,11,27^ Our findings add to the body of evidence for CGM use in type 2 diabetes and recommendations that 14 days of CGM data is effective at providing enough glucose data to enable review of glucose control to target lowering of HbA1c.^29–31^

With regard to safety, the few events relating to sensor insertion or wear were typical of medical adhesive use and similar to those observed in other cohorts.^10,32^

This was a pilot study and it has a number of strengths that should be highlighted. First, the choice of study population adds to the limited published evidence on the use of CGM in type 2 diabetes. Second, this is the first piece of work to include patients from primary and secondary care settings, therefore, study findings are not limited to a particular group of health care professionals. Third, ‘real-world’ glucose management was used regardless of location of care and there was no mandated treatment protocol. This lack of more directed glucose management may have also been a limitation as glycaemic benefits might have been observed earlier and with fewer sensor wears had an insulin titration algorithm been in place. It is possible that the observed changes in HbA1c for group C may have been due to additional clinical contact and dialogue alone and the changes observed were not related to Libre Pro use. However, this is unlikely to be the case given that these patients were recruited because of poor glycaemic control with previous clinical intervention failing to improve glucose levels.

Other limitations include the apparent strong study effect observed in the control group which is a concern, although it is unclear how best to tackle this in future professional CGM studies. Moreover, non-blinding of either participants or clinicians can be regarded as a drawback and is a recognised issue with device studies. Finally, the choice of TIR as the primary endpoint, may not have been ideal given the study effect and HbA1c is possibly a better overall glycaemic marker as it is less affected by short-term behavioural modifications.

## Conclusion

To summarise, use of professional flash technology in insulin-treated type 2 diabetes managed within primary and secondary care sites was associated with a significant reduction in HbA1c level and improved satisfaction with treatment. TIR and time in hypoglycaemia were unaffected. These data suggest that this novel professional flash sensor-based glucose monitoring technology has the ability to introduce clinically meaningful changes in HbA1c with a favourable safety profile when used in primary and secondary care settings.

## Supplemental Material

Ajjan_et_al_supp_appendix_5_July_2018 – Supplemental material for Reduction in HbA1c using professional flash glucose monitoring in insulin-treated type 2 diabetes patients managed in primary and secondary care settings: A pilot, multicentre, randomised controlled trial Click here for additional data file. Supplemental material, Ajjan_et_al_supp_appendix_5_July_2018 for Reduction in HbA1c using professional flash glucose monitoring in insulin-treated type 2 diabetes patients managed in primary and secondary care settings: A pilot, multicentre, randomised controlled trial by Ramzi A Ajjan, Neil Jackson and Scott A Thomson in Diabetes & Vascular Disease Research
