# Supplementary material for: Reduction in HbA1c using professional flash glucose monitoring in insulin-treated type 2 diabetes patients managed in primary and secondary care settings: A pilot, multicentre, randomised controlled trial
Source: Diab Vasc Dis Res. 2019 Jul 4;16(4):385–95. doi: 10.1177/1479164119827456 (PMC6613178; doi:10.1177/1479164119827456)
Supplement: Ajjan_et_al_supp_appendix_5_July_2018 – Supplemental material for Reduction in HbA1c using professional flash glucose monitoring in insulin-treated type 2 diabetes patients managed in primary and secondary care settings: A pilot, multicentre, randomised controlled trial [file Ajjan_et_al_supp_appendix_5_July_2018.pdf]

Supplementary material appendix:

Reduction in HbA1c Using Professional Flash Glucose Monitoring in Insulin-Treated Type 2 Diabetes Patients Managed in Primary and Secondary Care Settings: A Pilot, Multicentre, Randomised Controlled Trial

Ajjan R A, Jackson N[2] Thomson A S .

Table S1: Healthcare professional questionnaire responses

Indicate your level of agreement with the following statements:	Strongly Agree	Agree	Neither Agree or Disagree	Disagree	Strongly Disagree	No Response Provided
The reports are easy to understand	17 (36.2%)	23 (48.9%)	7 (14.9%)			
The reports are easy to read	17 (36.2%)	26 (55.3%)	4 (8.5%)			
The reports present the data in a clear way	18 (38.3%)	19 (40.4%)	9 (19.1%)	1 (2.1%)		
The reports allow me to **easily** determine how much time my patient's glucose levels are within target range	21 (44.7%)	22 (46.8%)	4 (8.5%)			
The reports allow me to **quickly** determine how much time my patient's glucose levels are within target range	19 (40.4%)	19 (40.4%)	8 (17.0%)	1 (2.1%)		
The reports help me to identify hypoglycaemic risk	27 (57.4%)	18 (38.3%)	1 (2.1%)	1 (2.1%)		
The reports allow me to **easily** see when my patient's glucose is in and out of target range	23 (48.9%)	22 (46.8%)	2 (4.3%)			
The reports allow me to **quickly** see when my patient's glucose is in and out of target range	19 (40.4%)	21 (44.7%)	6 (12.8%)	1 (2.1%)		
The reports help me **easily** see frequency, duration and pattern of ***hypoglycaemic*** events	18 (38.3%)	23 (48.9%)	5 (10.6%)	1 (2.1%)		
The reports help me **quickly** see frequency, duration and pattern of ***hypoglycaemic*** events	17 (36.2%)	21 (44.7%)	5 (10.6%)	4 (8.5%)		
The reports help me **easily** see frequency, duration and pattern of ***hyperglycaemic*** events	18 (38.3%)	22 (46.8%)	6 (12.8%)	1 (2.1%)		
The reports help me **quickly** see frequency, duration and pattern of ***hyperglycaemic*** events	17 (36.2%)	21 (44.7%)	8 (17.0%)	1 (2.1%)		
The reports visual presentation means I am able to effectively share information with my patients	22 (46.8%)	23 (48.9%)	2 (4.3%)			
The reports help me assess the need for therapy changes, such as dose adjustments	14 (29.8%)	24 (51.1%)	9 (19.1%)			
The reports help me target areas for therapy change, such as dose adjustments	15 (31.9%)	22 (46.8%)	10 (21.3%)			
The Glucose Pattern Insights table helps me **easily** see where I need to adjust therapy	12 (25.5%)	23 (48.9%)	11 (23.4%)	1 (2.1%)		
The Glucose Pattern Insights table helps me **quickly** see where I need to adjust therapy	11 (23.4%)	25 (53.2%)	10 (21.3%)	1 (2.1%)		
I would like to use this System with my Type 1 patients	27 (57.4%)	12 (25.5%)	5 (10.6%)			3 (6.4%)
I would like to use this System with my Type 2 patients	26 (55.3%)	12 (25.5%)	5 (10.6%)	1 (2.1%)	1 (2.1%)	2 (4.3%)
I would like to use this System with my Type 2 patients who use insulin	29 (61.7%)	10 (21.3%)	5 (10.6%)	1 (2.1%)		2 (4.3%)
I would like to use this System with **all** my patients who use insulin	28 (59.6%)	8 (17.0%)	6 (12.8%)	2 (4.3%)	1 (2.1%)	2 (4.3%)
It was **easy** to generate reports	15 (31.9%)	22 (46.8%)	7 (14.9%)	2 (4.3%)		1 (2.1%)
It was **quick** to generate reports	15 (31.9%)	17 (36.2%)	11 (23.4%)	3 (6.4%)		1 (2.1%)
Downloading the data from the sensor and generating reports fit into my normal clinic routine	7 (14.9%)	6 (12.8%)	19 (40.4%)	9 (19.1%)	3 (6.4%)	3 (6.4%)

Table S2: Primary care healthcare professional questionnaire responses

**Indicate your level of agreement with the following statements:**	**Strongly Agree**	**Agree**	**Neither Agree or Disagree**	**Disagree**	**Strongly Disagree**	**No Response Provided**
The reports are easy to understand	12 (32.4%)	19 (51.4%)	6 (16.2%)			
The reports are easy to read	12 (32.4%)	21 (56.8%)	4 (10.8%)			
The reports present the data in a clear way	14 (37.8%)	14 (37.8%)	8 (21.6%)	1 (2.7%)		
The reports allow me to **easily** determine how much time my patient's glucose levels are within target range	18 (48.6%)	17 (45.9%)	2 (5.4%)			
The reports allow me to **quickly** determine how much time my patient's glucose levels are within target range	15 (40.5%)	16 (43.2%)	5 (13.5%)	1 (2.7%)		
The reports help me to identify hypoglycaemic risk	23 (62.2%)	14 (37.8%)				
The reports allow me to **easily** see when my patient's glucose is in and out of target range	18 (48.6%)	18 (48.6%)	1 (2.7%)			
The reports allow me to **quickly** see when my patient's glucose is in and out of target range	13 (35.1%)	19 (51.4%)	4 (10.8%)	1 (2.7%)		
The reports help me **easily** see frequency, duration and pattern of ***hypoglycaemic*** events	16 (43.2%)	17 (45.9%)	3 (8.1%)	1 (2.7%)		
The reports help me **quickly** see frequency, duration and pattern of ***hypoglycaemic*** events	15 (40.5%)	15 (40.5%)	5 (13.5%)	2 (5.4%)		
The reports help me **easily** see frequency, duration and pattern of ***hyperglycaemic*** events	14 (37.8%)	19 (51.4%)	3 (8.1%)	1 (2.7%)		
The reports help me **quickly** see frequency, duration and pattern of ***hyperglycaemic*** events	13 (35.1%)	18 (48.6%)	5 (13.5%)	1 (2.7%)		
The reports visual presentation means I am able to effectively share information with my patients	15 (40.5%)	21 (56.8%)	1 (2.7%)			
The reports help me assess the need for therapy changes, such as dose adjustments	11 (29.7%)	19 (51.4%)	7 (18.9%)			
The reports help me target areas for therapy change, such as dose adjustments	11 (29.7%)	17 (45.9%)	9 (24.3%)			
The Glucose Pattern Insights table helps me **easily** see where I need to adjust therapy	9 (24.3%)	20 (54.1%)	7 (18.9%)	1 (2.7%)		
The Glucose Pattern Insights table helps me **quickly** see where I need to adjust therapy	8 (21.6%)	19 (51.4%)	9 (24.3%)	1 (2.7%)		
I would like to use this System with my Type 1 patients	21 (56.8%)	9 (24.3%)	4 (10.8%)			3 (8.1%)
I would like to use this System with my Type 2 patients	21 (56.8%)	9 (24.3%)	3 (8.1%)	1 (2.7%)	1 (2.7%)	2 (5.4%)
I would like to use this System with my Type 2 patients who use insulin	23 (62.2%)	7 (18.9%)	4 (10.8%)	1 (2.7%)		2 (5.4%)
I would like to use this System with **all** my patients who use insulin	22 (59.5%)	7 (18.9%)	4 (10.8%)	1 (2.7%)	1 (2.7%)	2 (5.4%)
It was **easy** to generate reports	10 (27.0%)	18 (48.6%)	6 (16.2%)	2 (5.4%)		1 (2.7%)
It was **quick** to generate reports	10 (27.0%)	15 (40.5%)	9 (24.3%)	2 (5.4%)		1 (2.7%)
Downloading the data from the sensor and generating reports fit into my normal clinic routine	4 (10.8%)	5 (13.5%)	16 (43.2%)	6 (16.2%)	3 (8.1%)	3 (8.1%)

Table S3: Secondary care healthcare professional questionnaire responses

**Indicate your level of agreement with the following statements:**	**Strongly Agree**	**Agree**	**Neither Agree or Disagree**	**Disagree**	**Strongly Disagree**
The reports are easy to understand	5 (50.0%)	4 (40.0%)	1 (10.0%)		
The reports are easy to read	5 (50.0%)	5 (50.0%)			
The reports present the data in a clear way	4 (40.0%)	5 (50.0%)	1 (10.0%)		
The reports allow me to **easily** determine how much time my patient's glucose levels are within target range	3 (30.0%)	5 (50.0%)	2 (20.0%)		
The reports allow me to **quickly** determine how much time my patient's glucose levels are within target range	4 (40.0%)	3 (30.0%)	3 (30.0%)		
The reports help me to identify hypoglycaemic risk	4 (40.0%)	4 (40.0%)	1 (10.0%)	1 (10.0%)	
The reports allow me to **easily** see when my patient's glucose is in and out of target range	5 (50.0%)	4 (40.0%)	1 (10.0%)		
The reports allow me to **quickly** see when my patient's glucose is in and out of target range	6 (60.0%)	2 (20.0%)	2 (20.0%)		
The reports help me **easily** see frequency, duration and pattern of ***hypoglycaemic*** events	2 (20.0%)	6 (60.0%)	2 (20.0%)		
The reports help me **quickly** see frequency, duration and pattern of ***hypoglycaemic*** events	2 (20.0%)	6 (60.0%)		2 (20.0%)	
The reports help me **easily** see frequency, duration and pattern of ***hyperglycaemic*** events	4 (40.0%)	3 (30.0%)	3 (30.0%)		
The reports help me **quickly** see frequency, duration and pattern of ***hyperglycaemic*** events	4 (40.0%)	3 (30.0%)	3 (30.0%)		
The reports visual presentation means I am able to effectively share information with my patients	7 (70.0%)	2 (20.0%)	1 (10.0%)		
The reports help me assess the need for therapy changes, such as dose adjustments	3 (30.0%)	5 (50.0%)	2 (20.0%)		
The reports help me target areas for therapy change, such as dose adjustments	4 (40.0%)	5 (50.0%)	1 (10.0%)		
The Glucose Pattern Insights table helps me **easily** see where I need to adjust therapy	3 (30.0%)	3 (30.0%)	4 (40.0%)		
The Glucose Pattern Insights table helps me **quickly** see where I need to adjust therapy	3 (30.0%)	6 (60.0%)	1 (10.0%)		
I would like to use this System with my Type 1 patients	6 (60.0%)	3 (30.0%)	1 (10.0%)		
I would like to use this System with my Type 2 patients	5 (50.0%)	3 (30.0%)	2 (20.0%)		
I would like to use this System with my Type 2 patients who use insulin	6 (60.0%)	3 (30.0%)	1 (10.0%)		
I would like to use this System with **all** my patients who use insulin	6 (60.0%)	1 (10.0%)	2 (20.0%)	1 (10.0%)	
It was **easy** to generate reports	5 (50.0%)	4 (40.0%)	1 (10.0%)		
It was **quick** to generate reports	5 (50.0%)	2 (20.0%)	2 (20.0%)	1 (10.0%)	
Downloading the data from the sensor and generating reports fit into my normal clinic routine	3 (30.0%)	1 (10.0%)	3 (30.0%)	3 (30.0%)	

Table S4: Sensor Insertion Site Symptoms Summary

Sign/ Symptom	Control Group A (N=52)		Intervention group B (2 reviews) (N=46)		Intervention group C (4 reviews) (N=50)		Not Randomised (N=27)		Total (N=175)	
	N (%) participants	N events	N (%) participants	N events	N (%) participants	N events	N (%) participants	N events	N (%) participants	N events
Total	5 (9.6%)	8	11 (23.9%)	16	12 (24.0%)	17	1 (3.7%)	1	29 (16.6%)	42
Mild	5 (9.6%)	8	10 (21.7%)	13	12 (24.0%)	15	0	0	27 (15.4%)	36
Moderate	0	0	3 (6.5%)	3	1 (2.0%)	2	1 (3.7%)	1	5 (2.9%)	6
Erythema	2 (3.8%)	2	6 (13.0%)	7	5 (10.0%)	7	0	0	13 (7.4%)	16
Mild	2 (3.8%)	2	6 (13.0%)	6	6 (12.0%)	7	0	0	14 (8.0%)	15
Moderate:	0	0	1 (2.2%)	1	0	0	0	0	1 (0.6%)	1
Itching	2 (3.8%)	2	4 (8.7%)	4	3 (6.0%)	3	1 (3.7%)	1	10 (5.7%)	10
Mild	2 (3.8%)	2	4 (8.7%)	4	3 (6.0%)	3	0	0	9 (5.1%)	9
Moderate	0	0	0	0	0	0	1 (3.7%)	1	1 (0.6%)	1
Bleeding	2 (3.8%)	2	1 (2.2%)	1	2 (4.0%)	2	0	0	5 (2.9%)	5
Mild	2 (3.8%)	2	1 (2.2%)	1	2 (4.0%)	2	0	0	5 (2.9%)	5
Bruising	2 (3.8%)	2	1 (2.2%)	1	1 (2.0%)	1	0	0	4 (2.3%)	4
Mild	2 (3.8%)	2	0	0	1 (2.0%)	1	0	0	3 (1.7%)	3
Moderate	0	0	1 (2.2%)	1	0	0	0	0	1 (0.6%)	1
Rash	0	0	1 (2.2%)	1	1 (2.0%)	3	0	0	2 (1.1%)	4
Mild	0	0	1 (2.2%)	1	1 (2.0%)	1	0	0	2 (1.1%)	2
Moderate	0	0	0	0	1 (2.0%)	2	0	0	1 (0.6%)	2
Pain	0	0	1 (2.2%)	1	1 (2.0%)	1	0	0	2 (1.1%)	2
Mild	0	0	1 (2.2%)	1	1 (2.0%)	1	0	0	2 (1.1%)	2
Oedema	0	0	1 (2.2%)	1	0	0	0	0	1 (0.6%)	1
Moderate	0	0	1 (2.2%)	1	0	0	0	0	1 (0.6%)	1

List of Investigators and Study Sites

Chief Investigator:

Dr Ramzi Ajjan, St. James University Hospital, Leeds, UK

Other secondary care investigators:

Prof Rudy Bilous, James Cook University Hospital, Middlesborough, UK

Dr Shanthi Chandran, Milton Keynes Hospital, Milton Keynes, UK

Dr Pratik Choudhary, Kings College Hospital, London, UK

Dr John New, Salford Royal Hospital, Salford, UK

Primary care investigators:

Dr Narayan Annamalai, Albany House Medical Centre, Wellingborough UK

Dr Matthew Capehorn, Clifton Medical Centre, Rotherham, UK

Dr Simon Cartwright, White Horse Medical Centre, Faringdon, UK

Dr Kevin Douglas, Claremont Medical Practice, Exmouth, UK

Dr Stephen Fowler, Friarsgate Practice, Winchester, UK

Dr Richard Gaunt, Rowden Surgery, Chippenham, UK

Dr Neil Jackson, Pound Hill Medical Group, Crawley, UK

Dr Ross Martin, Greenwood and Sneinton Family Medical Centre, Nottingham, UK

Dr Timothy Myhill, Rothwell Surgery, Rothwell, UK

Dr Carolyn Paul, The Kiltearn Medical Centre, Nantwich, UK

Dr Neil Paul, Ashfields Primary Care Centre, Sandbach, UK

Dr Rory Reed, Westongrove Partnership Bedgrove Surgery, Aylesbury, UK

Dr Asad Sabir, Omnia Practice, Birmingham, UK

Dr Raj Sharma, Sea Road Surgery, Bexhill, UK

Dr Gary Solomons, Parkwood Surgery, Hemel Hempstead, UK

Dr Christopher Strang, Mortimer Surgery, Mortimer, UK

Dr Scott Thomson, Atherstone Surgery, Atherstone, UK

Dr Tony Wright, Hathaway Medical Centre, Chippenham, UK
